# Meconium-stained Amniotic Fluid among Term Deliveries in a Tertiary Care Centre: A Descriptive Cross-sectional Study

**DOI:** 10.31729/jnma.7604

**Published:** 2022-08-31

**Authors:** Pawan Shakya, Manoj Kumar Yadav, Sagar Poudel

**Affiliations:** 1 Department of Surgery, District Hospital, Ramechhap Bazaar, Ramechhap, Nepal

**Keywords:** *apgar score*, *caesarean section*, *gestational age*, *perinatal death*, *pregnancy*

## Abstract

**Introduction:**

Although the precise aetiology of meconium-stained amniotic fluid is still unclear, risk factors include advanced gestational age at delivery, mode of delivery, the prolonged second stage of labour, and intrauterine infection. It has been associated with poor perinatal outcomes including low Apgar scores, increased incidence of neonatal intensive care admission, and a high rate of perinatal death. The objective of the study was to find out the prevalence of meconium-stained amniotic fluid in term deliveries in a tertiary care centre.

**Methods:**

A descriptive cross-sectional study was done among term deliveries in the Department of Obstetrics and Gynaecology, in a tertiary care centre from inpatient records starting from 29 November 2019 to 29 November 2020 after obtaining ethical approval from the Institutional Review Committee (Reference number: PMG1911281316). Convenience sampling was done. Point estimate and 95% Confidence Interval were calculated.

**Results:**

Out of 1699 term deliveries, meconium-stained amniotic fluid was seen in 91 (5.35%) (4.286.42, 95% Confidence Interval). Among these 69 (75.82%) newborns were delivered through lower segment caesarean section and 61 (67%) newborns had Grade II meconium-stained amniotic fluid.

**Conclusions:**

The prevalence of meconium-stained amniotic fluid was similar to the studies done in similar settings.

## INTRODUCTION

Meconium-stained amniotic fluid (MSAF) is a potentially serious sign of foetal compromise, and it is associated with increased perinatal mortality and morbidities.^1^ MSAF occurs in 5 to 20% of pregnancies, especially in term and post-term.^2^ Some factors associated with it are placental insufficiency, maternal hypertension, pre-eclampsia, oligohydramnios, or maternal drug abuse.^3^

Few studies have been done to determine the prevalence of MSAF delivery in Nepal. Identification of the grade of meconium will help to anticipate the need for neonatal resuscitation after deliveries, and preparedness with proper nursery and neonatal intensive care unit (NICU). It will eventually help to improve the neonatal outcome and reduce neonatal mortality and morbidity associated with MSAF.

The objective of this study was to find out the prevalence of MSAF in term deliveries in a tertiary care centre.

## METHODS

This descriptive cross-sectional study was conducted in the Department of Obstetrics and followed up in the post-partum ward, nursery and NICU of Patan Academy of Health Sciences (PAHS), Lagankhel, Lalitpur, Nepal. This study was conducted for one year, from 29 November 2019 to 29 November 2020. The data collection was started after the ethical approval from the Institutional Review Committee (Reference number: PMG1911281316). All babies born to women in labour who had completed more than 37 weeks of gestation were included. Babies born with presentations other than cephalic, twin pregnancies, stillbirth, and babies born to pregnant women with comorbidities such as hypertension, eclampsia, antepartum haemorrhage, and intrauterine foetal death were excluded from the study. Convenience sampling was done. The sample size was calculated using the following formula:


$$\begin{array}{ll} \text{n} & ={\text{Z}}^{2}\times \frac{\text{p}\times \text{q}}{{\text{e}}^{\text{2}}}\\ & =1.96^{2}\times \frac{0.0785\times 0.922}{0.02^{2}}\\ & =695 \end{array}$$

Where,

n= minimum required sample size
Z= 1.96 at 95% Confidence Interval (CI)
p= prevalence of MSAF, 7.85% ^8^
q= 1-p
e= margin of error, 2%

On doubling the sample size, it becomes 1390. However, final sample size taken was 1699. For written consent, a generic PAHS format in English (Annex-1) and Nepali (Annex-2) was used. The consent form contains all the information regarding the study, its objectives, methods, and aim of the study. Confidentiality of the research participants was maintained by not keeping a record of their names, specific addresses, and InPatient or Encounter numbers. A proforma sheet was filled by using the information derived from the interview of the subjects as well as from their medical records and from a thorough examination of pregnant women and newborn babies.

The gestational age was determined by ascertaining the first day of the last menstrual period. The consistency of meconium was divided into thin, moderate, and thick based on the expert opinion of Obstetricians. Intrauterine foetal heart rate (FHR) in all cases was noted. The mode of delivery of the baby was noted. Detailed information on foetal outcome was recorded and measured by Apgar scores in the first and fifth minutes, the requirement of neonatal resuscitation, development of Meconium Aspiration Syndrome (MAS) and need for admission to neonatal intensive care unit (Nursery and NICU). Birth asphyxia was diagnosed when the baby does not take spontaneous respiration at birth and Apgar scores at five minutes were less than seven.^4^ Baby if needed NICU admission was followed up in NICU and if admitted in the nursery it was followed up in the nursery and so on.

Data regarding age of mother, gestational age, grade of MSAF, mode of Delivery, Apgar score, the neonatal outcome of babies born through a meconium-stained amniotic fluid at first 24 hours of birth, respiratory status, NICU admission, need of ventilator support and mortality were collected and entered in Microsoft Excel 2013 and analysed in IBM SPSS Statistics 21.0. Point estimate and 95% Confidence Interval were calculated.

## RESULTS

Among 1699 term normal deliveries, the MSAF was found in 91 (5.35%) (4.28-6.42, 95% CI). A total of 69 (75.82%) were delivered via lower segment caesarean section (LSCS) (Table 1).

**Table 1:** Mode of delivery in MSAF (n= 91).

Mode of Delivery	n (%)
LSCS	69 (75.82)
Vaginal	22 (24.17)

**Figure 1:**
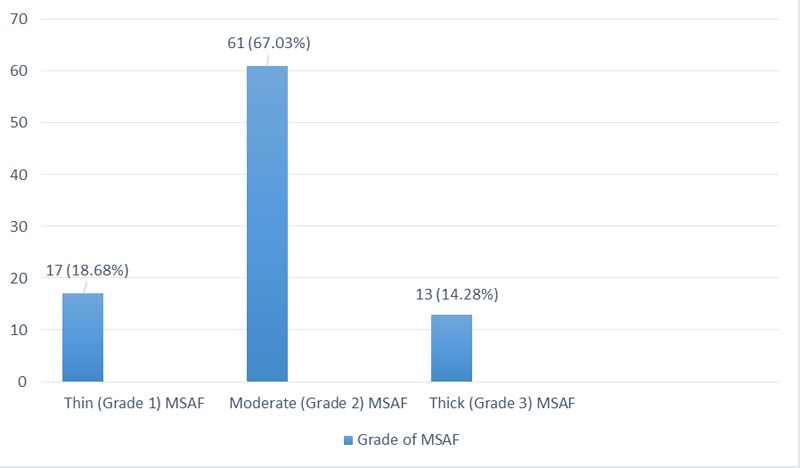
Grade of meconium-stained amniotic fluid (n= 91).

About two-thirds of cases 61 (67% had Garde II i.e.,moderate MSAF (Figure 1).

Only 2 (2.20%) babies developed MAS. A total of 57 (62.64%) babies cried immediately after birth whereas 34 (37.36%) of babies did not cry immediately after birth rather, they cried later or developed complications. Total 87 (95.60%) needed resuscitation either in the simple form of suction or bag and mask ventilation in some cases. Among the babies who developed respiratory issues within 24 hours, most of them 33 (36.30%) had nasal flaring followed by tachypnoea 6 (6.60%) and cyanosis 5 (5.50%). Very few 3 (3.30%) needed NICU admission within 24 hours rather nursery admission was found in 29 (31.90%) babies delivered by mothers with MSAF in their womb (Table 2).

**Table 2:** Neonatal outcome characteristics born with MSAF (n= 91).

Neonatal characteristics	n (%)
Baby cries	57 (62.60)
Need of resuscitation	87 (95.60)
**Respiratory issues**	
Nasal flaring	33 (36.30)
Cyanosis	5 (5.50)
Tachypnoea	6 (6.60)
None	47 (51.60)
Development of MAS	2 (2.20)
NICU admission	3 (3.30)
Nursery admission	29 (31.90)
Mortality	-

Out of 87 babies with MSAF who had received resuscitation, 73 (83.51%) had received airway clearance by suction, and 11 (12.16%) babies received bag and mask ventilation also. Most of the mothers i.e., 56 (61.50%) out of total MSAF were prepared for delivery during the daytime during office hours when there are senior faculties/doctors in their duties. Only 35 (38.50%) were delivered during the evening time or during the night. The Apgar score at 5 minutes is better than that at 1 minute. Apgar score at 1 minute was ≥7 in almost half of the cases 49 (53.80%) and almost half of the cases have Apgar less than 7 in 42 (46.20%). The median Apgar score was 7 at the first minute. At five minutes the Apgar score obtained was ≥7 among 88 (96.70%) cases. The Median Apgar score at five minutes is 8. The age category 20-34 years had the highest number of mother i.e. 81 (89%) with MSAF at the time of delivery. A total of 85 (93.40%) babies with MSAF were delivered by mothers with a gestational age of ≤40 WOG. Whereas, 6 (6.60%) babies with MSAF were of gestational age of >40 WOG.

## DISCUSSION

The prevalence of MSAF in term delivery in our study was 5.35%. This result was similar with the findings in Kavre 6.5% but lower than the findings of a study done in Ethiopia 17.85%.^5,6^ Among the mothers with MSAF, about three fourth of the delivery 75.8% was done surgically (LSCS) and only 24.2% of delivery was conducted via vaginal delivery, and this result is supported by most of the studies done where surgical management was done for such MSAF delivery cases. It was similar to the findings in Lahore and Larkana where about 85% of cases were delivered surgically and also in the study done in Kavre.^1,5^ These findings are opposite to the findings in Ethiopia where only 55.60% of MSAF were delivered by LSCS.^7^

The present study showed most of the mothers with MSAF, had a moderate grade of meconium 67% followed by thin meconium 18.7% and very few had thick meconium-stained amniotic fluid 14.3%, a similar finding was found in a study done in Karachi, where 76.5% had thin meconium.^8^ The outcome of our study is very similar to the study done in Ethiopia in 2019 where 65% had grade II or grade III meconium, and in India where most of the cases (56.20%) were grade II and grade III (30.70%) meconium-stained amniotic fluid.^7,9^ MAS is the most dreaded complication of MSAF with incidence ranging from 3-12% in babies born with MSAF.^10^ MAS increases admission rates in NICU, longterm neonatal morbidity and mortality, thus policies to thwart MAS need to be efficient, pragmatic, safe, and evidence-based. In our study, the result showed that almost all cases, except two cases 2.2%, did not develop MAS which is very similar to the study done in Kavre with 5.4% but opposing to the study done in Pakistan where 46% required resuscitation.^5,8^

Most of the babies delivered with MSAF cried immediately after birth; only 37.5% of cases did not cry just after delivery and some of them developed respiratory problems for 24 hours. Most (95.6%) of the baby delivered with MSAF, required resuscitation in the form of suction and very few required bag and mask ventilation 12.1% due to compromised airway probably due to MSAF. These findings are supported by the review articles of Siriwachirachai but contradictory to results obtained in a study done in Karachi, where very few (only 6.1%) had received resuscitation.^8,11^ In total, 3.30% required NICU admission, which was less compared to 30% in Patna, and 31.90% required nursery admission, contrary to 63% in a study in Pakistan.^8,12^ The mean Apgar score was 6 at 1 minute and 7 at 5 minutes, compared to 7 and 8 at 1 minute and 5 minutes respectively in a study done in Patna.^12^ No mortality was noted in our study whereas 0.4% and 3.03% in a study in Pakistan and Shillong respectively.^8,9^

The study was conducted in one centre only with a limited sample size within a limited time. The different grades of MSAF, Apgar scores, and decision for the mode of delivery have been conducted by different health personnel on duty creating variation in decision making and individual variations. The study was done during the COVID-19 pandemic, at a time when people might have feared visiting tertiary hospitals leaving space for bias.

## CONCLUSIONS

The prevalence of MSAF in term deliveries was similar to other study done in similar settings, but lower when compared to international studies. Whereas the modes of delivery and grades of MSAF were similar in frequency to the other reported literature.
